# Observations on the Relaxed Rectum

**Published:** 1845-04

**Authors:** H. Hunt


*Observations on the Relaxed Rectum.* By H. Hunt, M. D.—The author describes this as a malady of not unfrequent occurrence, and productive of much inconvenience and distress. The most prominent symptoms are obstinate constipation, a frequent desire to evacuate the bowels, a constant sensation of load in the rectum, which is not relieved by an evacuation, and the discharge, after much forcing, of mucus streaked with blood. The bladder, urethra, and other adjacent organs, often participate in the irritation.

On examination, the rectum will be found preternaturally enlarged, and more or less filled with large folds of mucous membrane pressing down on the anus, which impede the evacuation of the fæces, introduction of instruments, and injection of enemata.

This morbid condition of the mucous membrane the author attributes to a neglected state of the bowels, and repeated and great distension of the rectum by fæces, which causes the mucous membrane, when the bowel is empty, to hang in loose folds.

This disease, if neglected or mismanaged, gives rise to prolapsus ani, an irritable and painful state of the sphincter, and an introsusception of the upper and undilated portion of the intestine into the lower and dilated part.

The treatment recommended for the *simple* relaxed rectum is the avoidance of all aperient medicines, and the injection of a pint of cold water into the bowel every night previous to going to bed; the removal of the prolapsus, and the application of belladonna ointment to the irritable sphincter.

In the case of introsusception of the rectum, in addition to the use of the cold water injection, the exhibition of some mild aperient, taking care that whilst a costive or hardened state of the fæces is prevented, purging is avoided; and a course of the Hyd. c. Cretâ with hyoscyamus or conium, or of the iodide and sarsaparilla.

Dr. J. Johnson considered that the first class of cases described in the paper were common enough. The author, in these cases, seemed to deprecate altogether the use of aperient medicine; in this respect he, Dr. J., differed from him. It must be recollected that to effect a cure the colon as well as the rectum must be acted upon, as fæces often accumulated above the latter organ. Now the colon would not be reached by the small cold-water injection recommended in the paper, and these injections were far from being always harmless, for they were occasionally productive of great pain and severe tormina. He had even seen them producing faintness in a patient from these causes. In the treatment, then, of these affections, he preferred the milder aperients, such as tartrate of potash, or confection of senna, for these produced no irritation or straining; but were of the most essential service. In cases of relaxed and protruding rectum, he had used Ward's paste with much advantage. Under its employment the folds of the rectum became corrugated, and the canal altogether strengthened. With respect to local mechanical contrivance to keep the weakened bowel in position, he knew of none which in efficacy could be at all compared to two silk handkerchiefs, one of which was placed round the waist, and the other, attached to this behind and in front, kept a soft piece of sponge covered with linen in such a position that it pressed gently on the lower part of the rectum, and kept it in position.

Mr. Bransby Cooper bore testimony to the value of mild aperients in the cases mentioned by Dr. Johnson. In the treatment of prolapsed rectum, however, neither aperients nor mechanical contrivances of any kind were of half the service as the adoption of the plan recommended by Dr. Hunt in his paper—the evacuation of the bowels at night. When this plan was adopted the patient had the advantage of remaining in bed for seven or eight hours, during which time the rectum would be kept in position, and by this means a perfect cure might be effected; a termination rarely to be expected from the use of medicines or of mechanical support. The rule alluded to was one of great value in all diseases of the rectum. When the bowels were evacuated in the morning, irritation was kept up during the day, either by the sitting posture or by moving about, and all attempts at cure were frustrated.

Mr. Henry Lee stated that it appeared to him that the disease under consideration had hitherto been very imperfectly explained. In dissecting the rectum, a strong band of muscular fibres is found, arising from the posterior part of the pelvis, taking a course downwards and backwards, and encircling the lower extremity of the bowel. These muscular fibres are part of the levator ani, and although not usually described, are very important with regard to the disease in question. They lie in close contact with the sides and posterior part of the bowel, the lower two inches of which are thus encircled by strong muscular fibres, taking the same direction, and performing the same office, as the fibres of the sphincters ani. Whenever it happens that a portion of the relaxed mucous membrane comes within the grasp of these muscular fibres, the very natural effect is, that the return of the blood in the veins in the constricted part is retarded; and this in reality is the cause of the deep-seated dull pain, the tenderness, and the inflammation, which are so frequently observed in these cases. The principal object of local treatment in such instances is to relieve the inflamed portion of mucous membrane from the constriction caused by the muscular fibres, which has just been described. Temporary relief is immediately produced by returning the relaxed and congested mucous membrane to its natural station; but the benefit thus derived continues as long only as it is retained there.

It has been stated that the different instruments which have at various times been used in this complaint, instead of giving relief, have tended to increase the irritation of the part. This appears in a great measure, if not altogether, to depend upon the mode in which these instruments are constructed. They are generally so contrived that the bulb (by means of which the instrument is retained in the bowel) is placed, when the instrument is worn, immediately within the grasp of the muscular fibres of the levator ani, and it is to this cause that the irritation complained of is principally to be attributed. If, however, the stem of the instrument is made sufficiently long, so that the bulb, when introduced, is situated above instead of within the circular fibres of the levator ani, the instrument can be worn not only without inconvenience but often with the greatest comfort. Such instruments are kept by Messrs. Savigny, in St. James's Street. With regard to the plan proposed of removing portions of the mucous membrane in this disease, Mr. Lee considered the application of the strong nitric acid to the part as a safer and much less painful operation than that which was usually performed by means of a ligature.

Dr. Dickson supported the opinion of Dr. Johnson in regard to the employment of mild medicines, which acted on the bowel above the rectum, in case of obstinate constipation. He had found no remedy more efficacious in these cases than a combination of extract of hyoscyamus, and blue pill, in large doses. This was often of service in cases even of stricture situated high up in the bowel. He mentioned also with commendation a medicine for the knowledge of which he had been indebted to the late Dr. Gooch; it consisted of a mixture of aloes with sulphuric acid. In one instance in particular he had employed this medicine with the utmost advantage. The patient was a lady of constipated habit, who on one occasion had no movement of the bowels for sixteen days. Various gentler means to obtain an evacuation having been employed without effect, the aloes and sulphuric acid were administered; the bowels were quickly relieved, and during the remainder of her life, a period of six years, they were always easily acted upon. She died from diseases altogether independent of constipation. With respect to mechanical support in prolapsed rectum, he spoke with strong approbation of an instrument made by Mr. Laundy. The bowel by this apparatus was supported without inconvenience to the patient, and a cure by its means was occasionally completely effected.

Dr. Leonard Stewart related a case in which protrusion of the rectum had existed constantly for eight years, and occasioned much suffering. The patient was seized with apoplexy, for which bleeding and mercury were restored to. The functions of the bowel were completely restored under the influence of the salivation.

Mr. Macilwain considered that all the speakers, as well as the author, had taken too confined a view of causation in the diseases under discussion. The cause was more frequently in the portal circulation than in the rectum, and remedies applied to that part of the system would be found efficacious. This principle had been acknowledged, though not distinctly mentioned, in the recommendation of remedies whose chief action was on the liver. He spoke of the necessity of caution with respect to purgatives, which he had seen produce mischief in these affections.—*London Medical Gazette.*

